# Supplementary material for: HIVprotI: an integrated web based platform for prediction and design of HIV proteins inhibitors
Source: J Cheminform. 2018 Mar 9;10:12. doi: 10.1186/s13321-018-0266-y (PMC5845081; doi:10.1186/s13321-018-0266-y)
Supplement: Supplementary file 2 — Additional file 2. Source code of HIVProtI web server. [file 13321_2018_266_MOESM2_ESM.zip › HIVprotI_Source-code/hivprotI/weka-3-6-6/doc/weka/core/pmml/package-frame.html]

weka.core.pmml


weka.core.pmml

Interfaces 
  
*PMMLModel*

Classes 
  
BuiltInArithmetic
  
BuiltInMath
  
BuiltInString
  
Constant
  
DefineFunction
  
DerivedFieldMetaInfo
  
Discretize
  
Expression
  
FieldMetaInfo
  
FieldMetaInfo.Interval
  
FieldMetaInfo.Value
  
FieldRef
  
Function
  
MappingInfo
  
MiningFieldMetaInfo
  
MiningSchema
  
NormContinuous
  
NormDiscrete
  
PMMLFactory
  
PMMLUtils
  
TargetMetaInfo

Enums 
  
FieldMetaInfo.Interval.Closure
  
FieldMetaInfo.Optype
  
FieldMetaInfo.Value.Property
